# Hierarchy of hybrid materials. Part-II: The place of organics-*on*-inorganics in it, their composition and applications

**DOI:** 10.3389/fchem.2023.1078840

**Published:** 2023-01-25

**Authors:** Junnan Song, Anna S. Vikulina, Bogdan V. Parakhonskiy, Andre G. Skirtach

**Affiliations:** ^1^ Nano-BioTechnology Group, Department of Biotechnology, Faculty of Bioscience Engineering, Ghent University, Ghent, Belgium; ^2^ Bavarian Polymer Institute, Friedrich-Alexander-Universität Erlangen-Nürnberg, Bayreuth, Germany

**Keywords:** hybrid materials, organics, inorganics, nanoparticles, colloids, flat surfaces, modifications and applications

## Abstract

Hybrid materials or hybrids incorporating organic and inorganic constituents are emerging as a very potent and promising class of materials due to the diverse but complementary nature of their properties. This complementarity leads to a perfect synergy of properties of the desired materials and products as well as to an extensive range of their application areas. Recently, we have overviewed and classified hybrid materials describing inorganics-*in*-organics in Part-I (Saveleva, et al., Front. Chem., 2019, 7, 179). Here, we extend that work in Part-II describing organics–*on*-inorganics, i.e., inorganic materials modified by organic moieties, their structure and functionalities. Inorganic constituents comprise of colloids/nanoparticles and flat surfaces/matrices comprise of metallic (noble metal, metal oxide, metal-organic framework, magnetic nanoparticles, alloy) and non-metallic (minerals, clays, carbons, and ceramics) materials; while organic additives can include molecules (polymers, fluorescence dyes, surfactants), biomolecules (proteins, carbohydtrates, antibodies and nucleic acids) and even higher-level organisms such as cells, bacteria, and microorganisms. Similarly to what was described in Part-I, we look at similar and dissimilar properties of organic-inorganic materials summarizing those bringing complementarity and composition. A broad range of applications of these hybrid materials is also presented whose development is spurred by engaging different scientific research communities.

## 1 Introduction

The growth of research in the area of hybrid materials is continuing to be robust, benefiting from advanced processing methods (Lutich et al., 2009; Yang et al., 2019; Ariga et al., 2020), emerging technologies (Moreels et al., 2008; Zhu et al., 2021) and materials (Gaponik and Rogach, 2010; Yan et al., 2014; Zuaznabar-Gardona and Fragoso, 2018; Karthick et al., 2022), which facilitate tremendous growth and establish interconnections between associated scientific research areas and create new functionalities (Balazs et al., 2006; Christau et al., 2014; Leroux et al., 2015; Cui et al., 2016; Gao et al., 2016; Taubert et al., 2019; Cao et al., 2022; Stavitskaya et al., 2022). In the past, different approaches to the classification of hybrid materials have been discussed: those based on the interactions (Sanchez and Soler-Illia, 2006), wherein those associated with van der Waals, hydrogen bonding, and electrostatics are distinguished from those based on covalent and no-covalent bonds. On the other hand, distinction was made based on their composition (Kickelbick, 2003; Saveleva et al., 2019).

Recently and in the latter work, classification of “hybrid materials” was performed by Saveleva and co-authors, where modification of organic materials by inorganics was analyzed (Saveleva et al., 2019). It was emphasized that perfect complementarity of these two types of materials stemming from the fact that they are used to improve their respective properties or to bring-in additional functionalities into resultant hybrid materials (Ruiz-Hitzky et al., 2008; Wang et al., 2018b; Guo et al., 2020; Li et al., 2021a). A typically highlighted example is an assembly of hard and soft–two antagonist properties–materials which are different but complementary in regard with their properties. Peculiarly, such modifications have been used even from the time of ancient Greece (Sanchez et al., 2005) to particle-enhanced tires fabricated using carbon black, ZnO, MgS with rubber (Goodyear, 1856), extending to one a very practical material Bakelite produced by Leo Baekeland by admixing clays with resin (Baekeland, 1909). In contemporary research and development areas, functionalities and applications of hybrid materials continue to be extended, including, for example, water purification (Rigoletto et al., 2022) and antibacterial properties (Chen et al., 2022).

Hybrids were structurally and compositionally classified as follows (Saveleva et al., 2019):

i) Inorganic-modified organic materials (inorganics-*in*-organics);
ii) Organic molecule-modified inorganic materials (organics-*on*-inorganics), which can be sub-divided into:
  a) Colloids and nanoparticles functionalized by organic molecules;
  b) Inorganic structures modified and functionalized by organic molecules.

This review discusses the modification of inorganic materials and matrices by organic molecules, organics-*on*-inorganics (ii). The structure of these hybrid materials is discussed together with interactions of constituent blocks and applications. It thus provides the second part of the previously described Part-I by (Saveleva et al., 2019), which discussed inorganics-*in*-organics.

**FIGURE 1:**
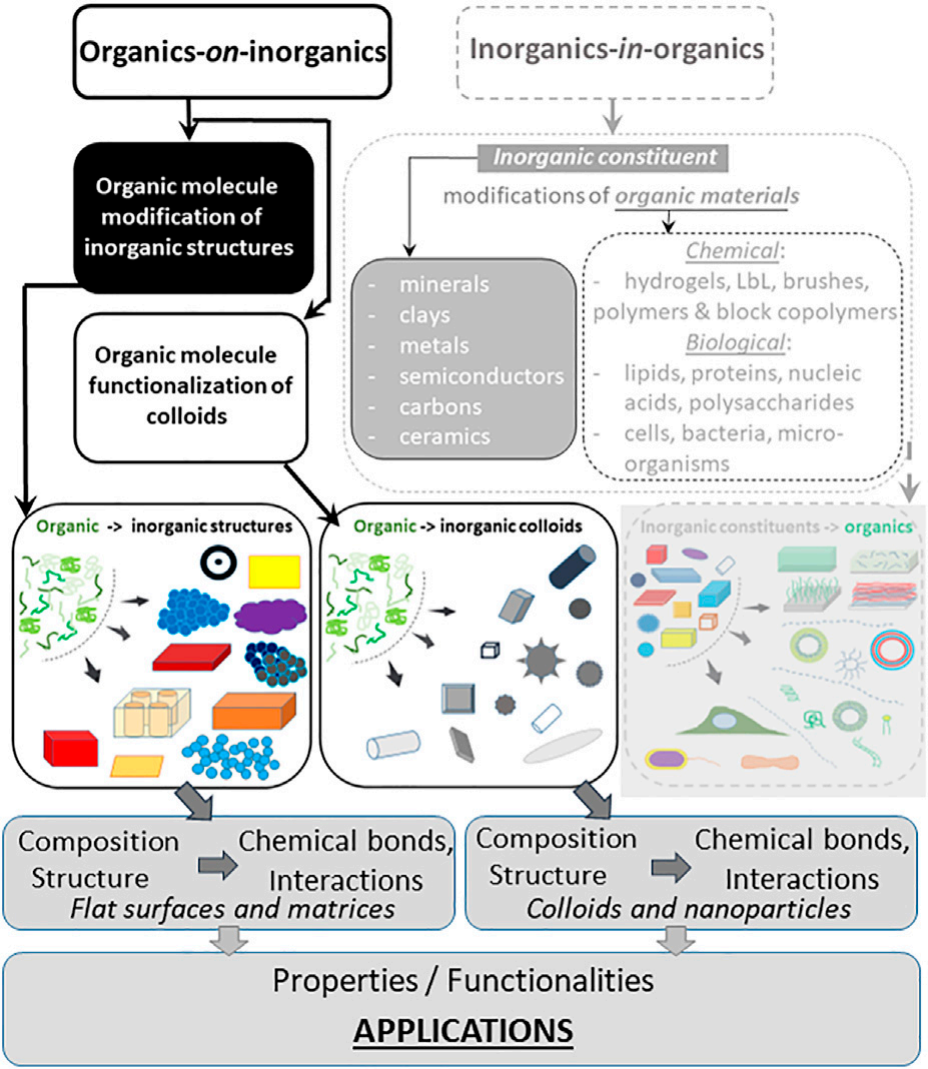
General classification of hybrid materials incorporating both organic and inorganic components. Adsorption of organic molecules on inorganic particles, structures, or matrices, referred to as organics-*in*-inorganics, is shown on the right-hand side in the grey-dashed rectangle constituted the Part-I of this classification (Saveleva et al., 2019). Functionalization of both inorganic colloidal particles and flat surfaces by organic molecules referred to as organics-*on*-inorganics, shown on the left-hand side, is the focus of this review. The bottom rows depict composition, chemical bonds, properties, and functionalities leading to development of a broad range of applications.

The hierarchy or classification of organics-*on*-inorganics is presented in Figure 1 and is extended analysing their composition, structure, chemical bonds, properties and functionalities, and applications. There, Part-I (Saveleva et al., 2019) is seen in grey rectangles (right-hand side), which introduced some modifications and functions of inorganics covering a range of materials from inorganic minerals, clays, metals, semiconductors, carbons, ceramics to organic ones: hydrogels, layer-by-layer (LbL) assembled polymer structures, brushes, and so on. Here, we focus on modifying inorganic particles, structures, and matrices with organic molecules–highlighted part (on the left-hand side) in Figure 1—and discuss their composition, interactions, and applications. Then, organic molecules and modifiers (organics) are introduced identifying the range of properties they can enable. Subsequently, we generally describe hybrid materials, summarize their properties, and provide conclusions and outlook.

## 2 Inorganic colloidal particle functionalization by organic molecules

**FIGURE 2:**
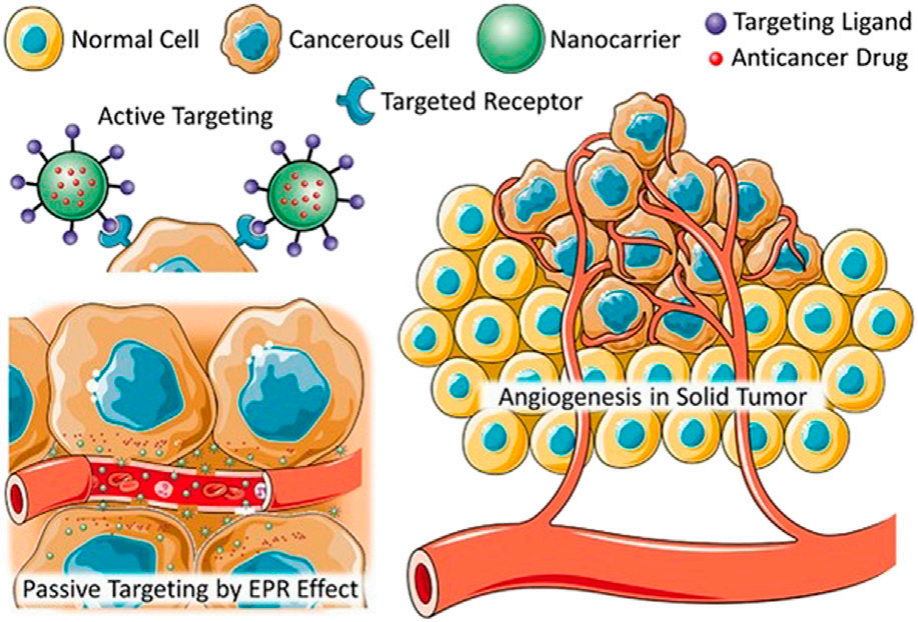
Schematic diagram of active and passive absorption of nanomedicines (Majumder and Minko, 2021), with the permission of the Wiley Online Library.

Developments in the area of colloidal particles has had a particularly profound impact in the field of biomedical applications including bioactive molecule sensing, targeted drug delivery, release systems, selective imaging agents, and diagnostics (Parakhonskiy et al., 2014; Tarakanchikova et al., 2020; Zhou et al., 2021). One of the reasons nanoparticles (NPs) are appealing for applications in biomedicine is because nano-size objects possess unique physicochemical properties, especially in a combination with an external electromagnetic field, which can mediate biological responses, for example, enhancing ^1^O_2_ generation (Sun et al., 2020a). Arguably, one of the most notable advantages brought by the NPs is the enhanced permeability and retention (EPR), which is considered to be a “golden standard” for designing new anticancer agents. Due to the EPR passive targeting and active endocytosis, cellular uptake (Figure 2) of nanocarriers could significantly improve anticancer/tumour drug efficiency and reduce their side effect and even rediscover drugs that may have tested failed clinically applications (Duan et al., 2019; McMahon et al., 2020; Peer et al., 2020; Majumder and Minko, 2021).

**FIGURE 3:**
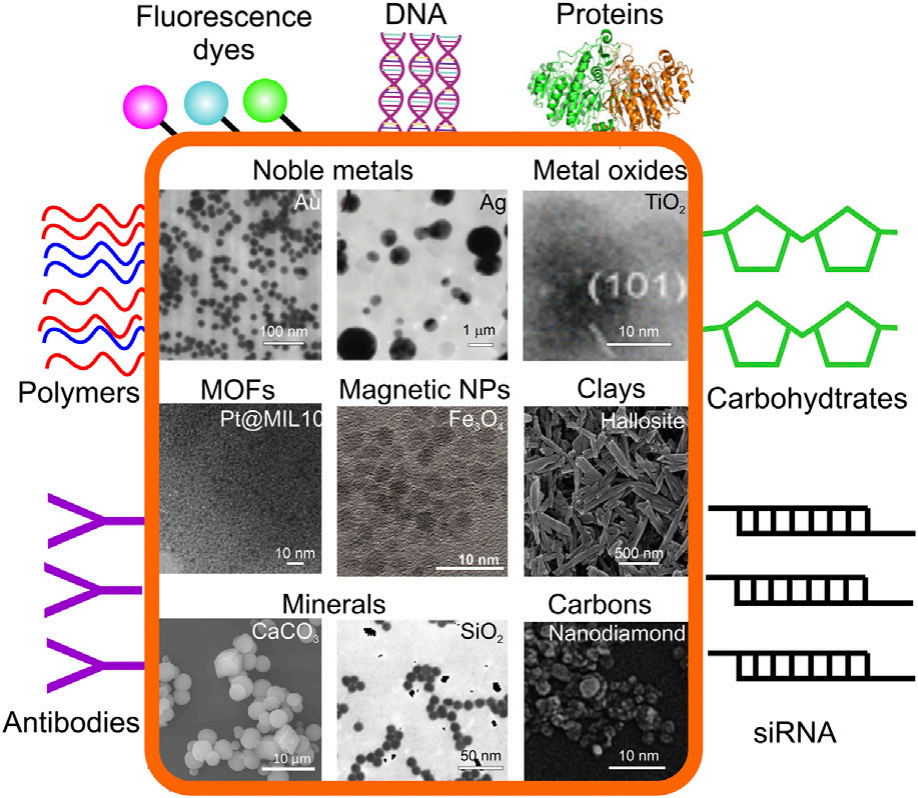
Organic molecules often used for inorganic NP functionalization in biomedical applications. The inorganic constituents (inside orange frame): noble metals (TEM images of Au NPs and Ag NPs reproduced from (Parakhonskiy et al., 2010), with permission from the RSC); metal oxides (TEM image of TiO_2_ NPS reproduced from (Sang et al., 2014) with permission from ACS); metal organic framework (MOFs; TEM image of Pt@MIL-101 reproduced from (Aijaz et al., 2012) with permission from ACS); magnetic NPs (TEM image of Fe_3_O_4_ reproduced from (Kozlova et al., 2020), with permission from MDPI); clays (TEM image of halloysite nanotubes reproduced from (Lvov et al., 2008), with permission from ACS), minerals (SEM image of CaCO_3_ microparticles reproduced from (Abalymo v et al., 2022), with permission from Elsivier, TEM image of SiO_2_ reproduced from (Ung et al., 2001), with permission from J. Phys.Chem.B), diamonds (TEM image of nanodiamonds reproduced from (Smith et al., 2009) with permission from Wiley-VCH. Organic molecules used for functionalization of inorganic particles and nanostructures are shown outside of the orange frame.

**FIGURE 4:**
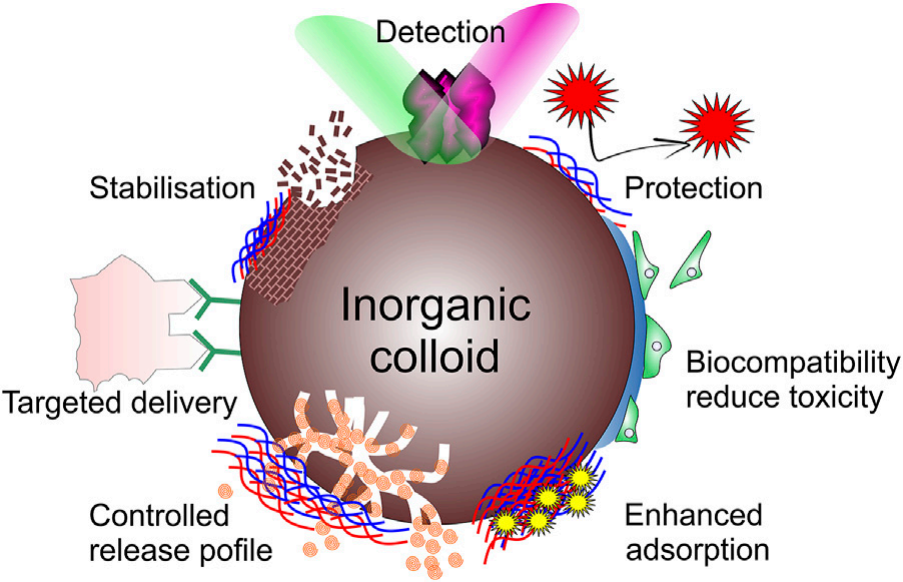
Functionalities provided by organic molecules adsorbed on inorganic NPs.

Compared with organic NPs, inorganic NPs’ inherent unique conductivity, magnetism, optical properties and variety in size, structure, and shape have become the hot spot in therapeutic diagnosis (Tan et al., 2018; Wang et al., 2018a; Sun et al., 2020b), whereas one of the broadest areas of research applying organic NPs is drug delivery. For example, superparamagnetic NPs can enhance magnetic resonance imaging (MRI) contrast, Au NPs are widely used in catalysis, drug delivery, imaging and photothermal therapy (Wang et al., 2006; Cui et al., 2014; Xiong et al., 2014; Fraire et al., 2019), while carbon quantum dots (CQDs) have inspired development of numerous applications particularly in biosensing and bioimaging due to their optical and fluorescence emission properties (Lim et al., 2015). Further, Figure 3 summarizes inorganic colloidal structures often functionalized with denoted organic molecules. Figure 4 summarizes applications of inorganic colloids modified by organics, particularly relevant in biomedical applications, and Table 1 summarizes selected applications of hybrid colloidal particles, where biomedicine is identified to be one of the most widely used application areas.

**TABLE 1:** The overview of the studies of hybrid organics-on-inorganics colloidal and their composition, feature/functionalities.

Composition of hybrid colloids			Functionalities and applications
Inorganic NPs		Organic molecules and reference	
Noble Metal	Au	4-(dimethylamino)pyridine (DMAP) REF Fraire et al (2019), polydimethylsiloxane (PDMS) Zhu et al. (2021), catalytic hairpin assembly Song et al. (2021), nucleic acid hybridization, aptamer-target binding, antigen-antibody recognition, enzyme Hua et al. (2021)	Selective and sensitive identification
		Layer-by-layer, PEI Lee et al. (2011), high-density lipoprotein Shen et al. (2018) CpG oligodeoxynuleotides Duan et al. (2019) Hyaluronic acid (Ha) (Sanfilippo et al. (2020) PEG, poly-l-lysine (PLL) Oladimeji et al. (2021)	Targeted delivery
		l-lysine Volodkin et al. (2009)	Remote release
		Gallic acid Kim et al. (2017), hyaluronic acid Sanfilippo et al. (2020)	Reduction of cytotoxicity
	Au, Ag	Conventional surfactants and ligands (PVP, CTAB, Citrate), polyol (EG, PEG) Zhang et al. (2011); Kang et al. (2019), thiol (-SH), amine (−NH2),carboxyl (−COOH), phosphine (−PR3), PEI Heuer-Jungemann et al. (2019); Kang et al. (2019); Roberts et al. (2020); Song et al. (2021)	Morphology guide
		Polymer (PEG) (Kang et al. (2019), Citrate Patungwasa and Hodak (2008); Wen et al. (2020) Cetyltrimethylammonium bromide (CTAB) Heuer-Jungemann et al. (2019)	Stabilization preservation and improvement of plasmonic particle functionalities
		DNA, protein Kang et al. (2019), Ferritin Moglia et al. (2021), pMPC King and Fiegel (2022)	Enhancement of biocompatibility, programmable assembly
	Ag	Chitosan (CTS), Polyvinylpyrrolidone (PVP) (Zhang et al. (2012a) Pomegranate rind extract Panáček et al. (2018), Starch nanofiber mats Lv et al. (2021), Mentha pulegium extract Wang and Wei (2022)	Stabilization
		Melamine (1,3,5-triazine-2,4,6-triamine) Li et al. (2019)	Label-free detection for targeted DNA delivery
		Bisphosphonate alendronate Benyettou et al. (2015), folic acid Yang et al. (2021a)	Targeted delivery
		Citrate, PVP Gitipour et al. (2016) Starch nanofiber mats Lv et al. (2021)	Reduction of cytotoxicity
	MXene (Ti3C2Tx)-Ag	Citrate, PDDA Liu et al. (2021)	Efficient and quantitative SERS biosensor platform
Metal Oxide	Bi_2_O_2_CO_3_-Cu_2_O	Silk fibroin Ji et al. (2020a)	Recyclable photocatalysts with enhanced photocatalytic activity, reduction of cytotoxicity
	ZnO	Reactive cyclic oligosaccharide: monochlorotriazinyl-β-cyclodextrin (MCT-β-CD), PEA Abdolmaleki et al. (2014)	Stabilization (good dispersion and less aggregation)
		Octylamine trioctylphosphine oxide, hexadecylamine, dodecylamine Heuer-Jungemann et al. (2019)	Surfactant morphology guide
		Polymer Zhao et al. (2017)	pH-triggered drug-delivery system
	TiO_2_	3-aminopropyltriethoxysilane (APTES), glutaraldehyde (GLU), laccase Hou et al. (2014)	Increasing catalytic activity, thermal and operational stability
	CuO	Protease and amylase Murugappan and Sreeram (2021)	
	MnO_2_	Macrophage Li et al. (2021b)	Good adsorption to target cells
	Al_2_O_3_, SiO_2_	Polyurethane (PU) Xavier (2021)	Enhancing anticorrosion
Metal-Organic Framework	MIL-101(Fe)-NH_2_	Long-chain polyamines (ethylenediamine, 1,2-bis(3-aminopropylamino)ethane Almáši et al. (2018)	Improving antibacterial, and biocompatible property
	Zr MOF UiO-66	α-cyano-4-hydroxycinnamic acid (α-CHC), Alendronate Abanades Lazaro et al. (2020)	
	MIL101 NPs	Zwitterionic hydrogel layer Zhou et al. (2021)	
	Cu-based MOFs	Pectin nanofibers polyethene oxide (PEO), folic acid Kiadeh et al. (2021)	
	NiCo_2_O_4_, Au	Nafion/thionine Li et al. (2011)	
Magnetic nanoparticles	Fe_3_O_4_, SiO_2_	Poly (ethylene glycol) (PEG), antibody Liu et al. (2020b)	Target delivery
	Fe_3_O_4_, SiO_2_	Poly (allylamine hydrochloride) (PAH) Shi et al. (2011)	Enhanced biocompatible, reduction of cytotoxicity
	Fe_3_O_4_	Polyethylene glycol, polypyrrole Song et al. (2014)	Stabilization, target cell tumour uptake
	Ultra-small superparamagnetic Fe_3_O_4_	Polydopamine, GE11 peptide Yang et al. (2019)	Encapsulation drug carriers
	Fe_3_O_4_, ZrO_2_	Glucose oxidase Haskell et al. (2020)	Controlling catalytic performance and stability
	Fe_3_O_4_	Hyaluronic acid Sun et al. (2021b)	Good adsorption to multiple types of immune cells
	Fe@Au Janus nanoparticles	Polyvinylpyrrolidone (PVP) Espinosa et al. (2020)	Magnetically guided and thermally activated cancer therapy
Minerals	CaCO_3_	Tritc-dextran Parakhonskiy et al. (2013), fluorescent dye Rhodamine 6G Parakhonskiy et al. (2012a) catalase, insulin, aprotinin Feoktistova et al. (2020)	Detection and release functionalities
		Alginate hydrogel Muderrisoglu et al. (2018)	Loading of active enzymes
		PAH, PSS Sergeeva et al. (2015)	
	Ca_3_(PO_4_)_2_	Poly acrylic acid (PAA) Wang et al. (2017)	pH-responsive drug-release vehicles
	Hydroxyapatite	Pam78 Zaheer et al. (2006)	NIR detection by bisphosphonate derivative
		Poly (ε-caprolactone) (PCL) Ji et al. (2020b)	Improving cytocompatibility and bio-compatibility
	Hydroxyapatite, Wollastonite (WST)clay	Potato starch (C_6_H_10_O_5_)n, Prabakaran et al. (2021)	
	SiO_2_, Au	Perfluoropentane Li et al. (2018a)	Stimuli-responsiveness, drug delivery and controlled release
	SiO_2_	CD-PGEA Zhang et al. (2017)	
		Hyaluronic acid Gupta et al. (2018)	
		Pyromellitic dianhydride (PMDA) Feng et al. (2019)	
		Poly (ethyleneglycol) N-(3-triethoxysilylpropyl)diethanolamine Climent and Rurack (2021)	Fluorescence imaging
Clay	Halloysite	Hexadecyl trimethy-lammonium bromide (HDTMA) Tan et al. (2016)	Stabilization
		Alginate and chitosan Lisuzzo et al. (2019)	pH-responsive drug-release vehicles
		Polyethyleneimine (PEI), polystyrene sulphonate (PSS) Lvov et al. (2008); Tan et al. (2016)	Drug release
	Laponite	Proteins, polymers Gaharwar et al. (2019)	Targeted delivery wound healing, tissue adhesive
Semiconductor	CdSe/ZnS, CdSe/CdS/ZnS	Polyethylene glycol Sukhanova et al. (2022)	Reduction of cytotoxicity
Carbons	Multi-walled carbon nanotubes (MWCNTs)	Hydrophilic moieties (MWCNT-OH, -COOH, -NH2, -SH) Kaufmann et al. (2017) Horseradish peroxidase, Ab2 (secondary antibody) BMIM·BF4 Cai et al. (2011)	Specific anti-oncogene detection drugs carriers
		Amphiphilic polycations (Poly (N-cetyl-4-vinylpyridinium bromide-co-N-ethyl-4-vinylpyridinium bromide-co-4-vinylpyridine)) (Sinani et al. (2005)	Modification of aqueous dispersions
	Carbon quantum dots (CQDs)	Glucose oxidase, boronic acid, bis(3-pyridylmethyl)amine, β-cyclodextrin Huang et al. (2016); Feng and Qian (2018) folic acid Choi et al. (2014)	Bioimaging with a high specificity
	Fluorescent nanodiamond (FNDs)	Glycosaminoglycans, Viral Envelope Proteins Pham et al. (2017)	Targeted delivery and imaging
	Fullerenes (C_60_)	Cyclodextrins Ikeda (2013)	Water-soluble fullerenes, photoinduced energy- and electron-transfer
	Carbon nano onions glassy carbon electrodes (GCE)	Polydopamine (PDA) Zuaznabar-Gardona and Fragoso (2018)	pH detection with a high detection sensitivity over the pH range of 2–10
	Graphene oxide (GO)	PEG Xu et al. (2014) poly (acrylamide) (PAA) (Xu et al. (2016)	Reduction of cytotoxicity, improving drug delivery efficiency
	Carbon nano onion clusters	PEI, PEG Sun et al. (2019)	Improving photothermal conversion efficiency

### 2.1 Stabilization

#### 2.1.1 The meaning and theory behind colloidal stabilization

**FIGURE 5:**
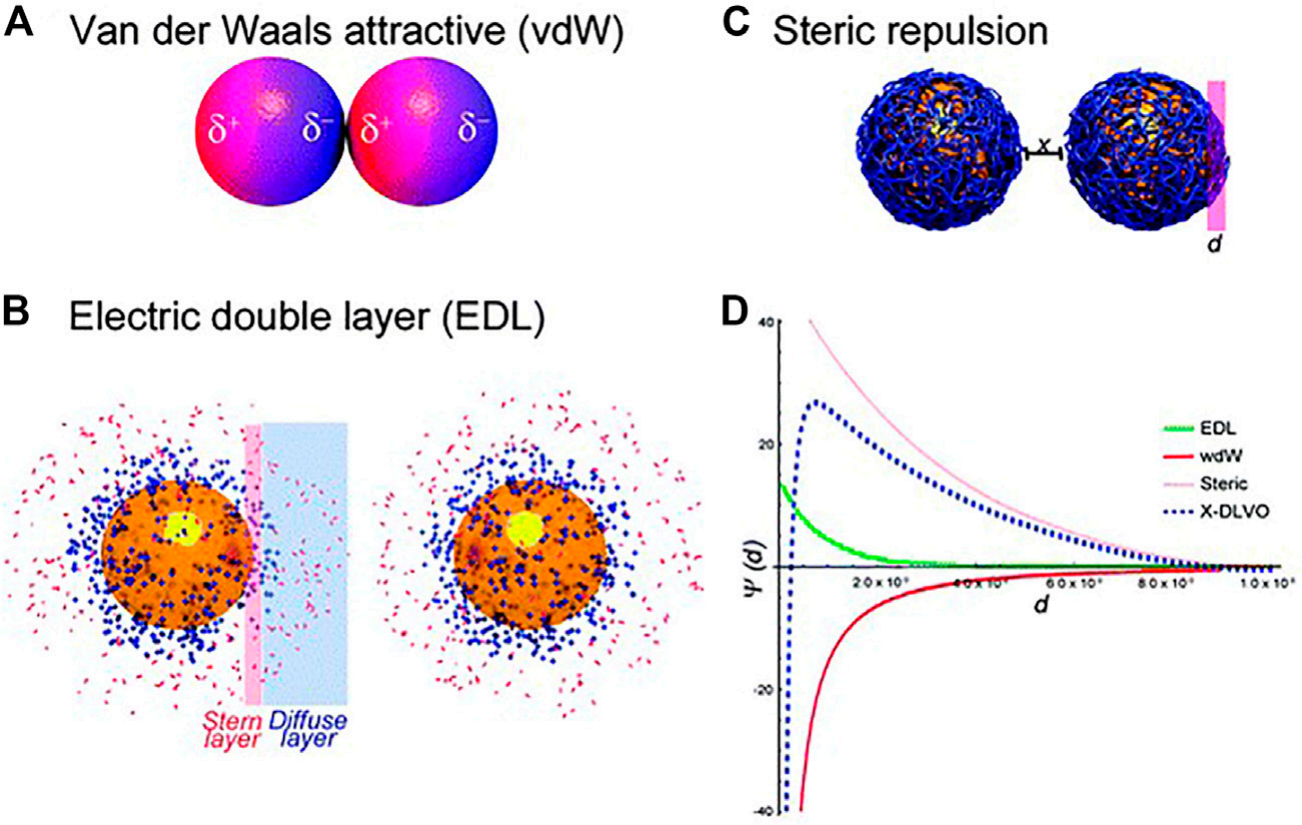
Schematics of colloidal interactions **(A–C)** and illustration of potential energy-distance curves describing colloidal interactions **(D)** (Moore et al., 2015).

Colloids are referred to as a system consisting of nanoparticles with the sizes from 10 nm to ∼1 μm dispersed in a fluid, most frequently the liquid phase. The Derjaguin–Landau–Verwey–Overbeek (DLVO) theory can quantify effectively the colloidal stability, but it can only work for aqueous solutions containing simple electrolytes. Briefly, the balance of attractive van der Waals (vdW) (Figure 5A) and repulsive forces caused by the electrostatic double layer (EDL) (Figure 5B) determines the colloidal stability in an aqueous suspension. Actually, besides vdW and EDL, steric repulsion (Figure 5C) inherent to macromolecules such as synthetic polymers or proteins could dramatically enhance the colloidal stability due to osmotic pressure and elastic recoiling effects (de Gennes, 1987). Of course, there are other forces needed to be taken into consideration, like depletion forces and magnetic forces controlled by an extra electromagnetic field (Petry et al., 2019; Espinosa et al., 2020). The study showed that fullerenes can be water-soluble and have a high stability achieved by host-guest interactions with cyclodextrins (Ikeda, 2013). It should be noted that taking into account the energy-distance curves, it would be possible to predict the behaviour of colloids. One example, aqueous solutions containing simple electrolytes, is shown in Figure 5D.

Despite significant breakthroughs in biomedical applications of nano- and micro-particles, the gap between research and their translation into clinics is still significant (Venditto and Szoka Jr, 2013). One of the primary challenges is the complexity of the system, while one should not exclude the lack of understanding of all mechanisms governing particle interactions and effects on biological objects, especially when they are immersed in a complex cellular environment (Moore et al., 2015). In part, this is because the actual system and environment including cell surface, extracellular matrix, and cell culture medium are quite complex compared to commonly used cell culture media like DMEM, MEM, and PBS solution. Most of the particles are taken up *via* active endocytosis, during which they are exposed to different conditions, like pH changes from 7.4 (in the extracellular medium) to 5.5 (in late acidified endosomes) to 4.5 (in endolysosomes). Moreover, endolysosomes are rich in hydrolytic enzymes that can affect/dissolve the entire NPs or their coatings. Internalized AgNPs were reported to degrade quickly into Ag^+^ when immersed in endolysosomes (Foldbjerg et al., 2015). Besides, behaviour of particles is sensitive to the ionic strength of the solution. For example, calcium carbonate (CaCO_3_) can recrystalize in hydroxyapatite (Ca_10_(PO_4_)_6_(OH)_2_) in cell medium, which contains the PO_4_^3−^ groups or aggregate in the presence of sodium/chloride ions (Na^+^/Cl^−^) (Parakhonskiy et al., 2012b) as well as crystalize and dissolve at relatively acidic pH values inside the cell (Abalymov et al., 2018).

Affected by the dynamic complex nature of the cellular environment, the loss of colloidal stability of particles would likely lead to colloidal aggregation into larger and irregularly shaped clusters and even dissolve entirely or stimulate recrystallisation or structural changes. The poor stability of colloids would lead to misrepresentative results or low reproducibility and, at the same time, influence their biodistribution, pharmacokinetics and systemic toxicity (Moore et al., 2015). Another problem is the corona effect which requires additional stabilization. This is because when nanoparticles are injected into the intravenous stream, the blood’s proteins would adsorb on the surface of the particles, thus forming a protein corona layer (Lee, 2021). Protein corona may lead to undesirable effects, for example, facilitating or inhibiting cellular uptake and mitigating or stimulating the immune response (Del Pino et al., 2014; Mosquera et al., 2018; Liu et al., 2020a). To guarantee that nanoparticle functions remain as stable as possible, organic molecules like PEG (Poly (ethylene glycol)), PAA (Poly (acrylic acid)) (Tambunlertchai et al., 2017; Xiao et al., 2020), nanocelluloses (Kaushik and Moores, 2016) are commonly reported to be added as stabilizing agents during the formation process.

#### 2.1.2 Approaches to stabilize inorganic colloids

One of the most widely and effectively accepted strategies to enhance colloidal stability is to avoid particle aggregation *via* surface modification (Albanese and Chan, 2011), involving for example steric and electrostatic stabilization (Bihari et al., 2008; Liu et al., 2021). Table 2 juxtaposes these three methods used to stabilize the inorganic particles illustrated with respective organic molecules.

**TABLE 2:** The methods used to stabilize inorganic colloids *via* organic molecules.

Stabilization method	Inorganic NPs	Organic molecule	Medium	Reference
Electrostatic interaction	Au, Ag	Citrate coating	DMEM + 10%FBS, water	Casals et al. (2011); Wen et al. (2020)
	CaCO_3_	Vitamin D3	Edible oil-in-water Pickering emulsion	Guo et al. (2021)
	SiO_2_	N-(6-aminohexyl)-3-aminopropyltrimethoxy silane	PBS, TRIS buffer, DMEM + 10%FCS	Graf et al. (2012)
Steric	Au	Thiolated PEG, amphiphilic block co-polymer (PVA-COOH)	Phosphate buffer with lysozyme, water	(Zhang et al., 2009; Wen et al., 2020)
		Gallic acid	2.5 mM glucose	Kim et al. (2017)
	SiO_2_	Poly (methyl methacrylate) (PMMA)	1-alkyl-3-methylimidazolium-based ionic liquids	Singh et al. (2019)
		PEG	TRIS buffer, DMEM + 10%FCS	Graf et al. (2012)
	Perovskites	hydrolyzed poly (methyl methacrylate) (h-PMMA), poly (ethylenimine) (PEI-25K)	Polar solvents (methanol)	Jiang et al. (2021)
Electrostatic	Au	Thiolated DNA	0.1 M NaCl	Mirkin et al. (1996); Lee et al. (2021)
		PEI/siRNA/PEI	10 Mm NaCl	Elbakry et al. (2009)
		PMPC (poly methacryloyloxyethyl phosphorylcholine)	Serum and lung lavage fluid	King and Fiegel, (2022)
	CeO_2_, γ-Fe_2_O_3_	PAA	DMEM, RPMI-1640 + 10%FBS	Chanteau et al., (2009); Safi et al., (2011)
	Au, Ag, Fe_3_O_4_, CoO, CeO_2_	FBS coating (10%)	complete cell culture medium (cCCM)	Casals et al. (2011)
	Fluorescent nano diamond	Protein polymers C4−K12 (polypeptide)	NaCl solution (at least 1 M)	Zheng et al. (2017)
	CdTe, CdSe/ZnS	chitosan	Queous solution (pH 5.6)	Slyusarenko et al. (2019)
	CaCO_3_	ALP, Alginate	PBS, 0.9 M NaCl	Abalymov et al. (2020)

##### 2.1.2.1 Electrostatic interaction

When NPs are exposed to a cell medium, colloidal interactions take place at the surface of particles (in the surrounding medium). The overall ionic strength of the new-mixture system determines the EDL fate. Typically, low ionic strength enlarges ion clouds extending further from the particle surface, which diminishes or resists particle-particle interaction. In contrast, an environment with a high ionic strength will suppress the EDL and enforce the attractive vdW leading to NP aggregation (Edwards and Williams, 2004). Electrostatic stabilization is generally reported to be poor in cell culture media (Moore et al., 2015).

##### 2.1.2.2 Steric stabilization

Steric stabilization is the universal method to reinforce colloidal stability. This is often achieved *via* natural macromolecules (e.g., chitosan, alginate) or synthetic polymer coatings, e.g. PVA (polyvinyl alcohol), PEG (polyethene glycol) (Zhang et al., 2009). Based on numerous studies, there are two ways to make the system more stable: a) coat the NPs with an uniform monolayer of macromolecules, which could provide extra robust steric stabilization, and b) prevent the proteins from attaching to NPs surfaces, which paves the way for bridging effects and leads to aggregation. This is because proteins contained in the cell culture media could cover the surface of particles forcing proteins to interact with more than one NP and facilitate aggregation (Biggs et al., 2000). It has been also shown that the effects of PEG molecules on AuNPs’ colloidal stabilization depend on their molecular weight and concentration (Zhang et al., 2012b).

##### 2.1.2.3 Electrostatic stabilization

One approach to improve colloidal stability is electrostatic stabilization which combines the effects of electrostatic and steric stabilization. In this case, macromolecules strongly coat the NP surface as a physical barrier and therefore impede proteins attaching to NPs’ surfaces. Meanwhile, these charged macromolecules would provide repulsive static electricity given that the environment of the cell culture medium is usually weak alkaline and negatively charged; the charge on NPs can be negative through functionalization by, for example, poly (acrylic acid) (PAA) (Chanteau et al., 2009) or it can be positive (for example, DMAP).

It should be noted that the interactions between the colloidal particles and the capping agents are sophisticatedly complex and depending on media, NPs, functional ligands, iron strength, *etc*. To interpret and mimic the relation of the colloidal and biological media precisely *in vitro*, there is still significant needs and that largely depends on interdisciplinary efforts to be made in materials science, cell biology, pharmaceutics, biophysics, *etc*.

### 2.2 Controlled loading and release

One of the most feasible ways to enhance the drug efficacy is to load a suitable amount of drug and release it at the lesions. The hybrid nanocomplex is a powerful tool to modify drug loading and optimize release behaviour (Li et al., 2017). The porous inorganic particles are frequently used as drug carriers. Functionalizing them with additional polymer layers improves the stability and contributes to controlled release mechanisms. For example, the functionalisation of porous CaCO_3_ particles with polymer layers would stabilize the containers on hour-, week-, even months scales, leading to a prolonged drug release. APTES-modified halloysites have a higher loading capacity and were shown to prolong the release of ibuprofen (Tan et al., 2016). The drug delivery system release behavior based on metal-organic framework (MOF) like MIL-101(Fe)-NH2 could be modified by long-chain polyamines (Almáši et al., 2018). Zhou *etc.*, designed a pH-triggered self-unpacking capsules consisting of a MOF and zwitterionic hydrogel coating (Zhou et al., 2021). The MOF core with a large pore size and surface guarantees high loading efficiency. The outer layer of hydrogel is made of pH-sensitive poly-Eudragit L100-55, which would dissolve at a pH > 5.5 and therefore protect the oral peptide drug from degradation when passes through an acidic gastrointestinal environment and is released in the intestinal fluid. Multi-walled carbon nanotubes functionized by hydrophilic organics might be explored as an efficient nanotransporters for oligodeoxynucleotide (Kaufmann et al., 2017). The nanohybrid graphene oxide (GO)-PEG/paclitaxel (PTX) shows relatively high loading capacity for PTX (11.2 wt%) and existed significantly higher cytotoxicity to human lung cancer A549 and human breast cancer MCF-7 cells compared to free PTX (Xu et al., 2014). Loading and release may differ for various drug deivery carriers, and in this regard polyelectrolyte multlayer nano- and micro-capsules are attractive carriers (Li et al., 2022b), which are composed of polyelectrolytes (organic molecules), but their functionalization with such inorganic particles as gold (Skirtach et al., 2005) or silver (Skirtach et al., 2004; Radziuk et al., 2007) allows to perform release.

### 2.3 Reduction of cytotoxicity and improvement of biocompatibility

There have been reports on drugs providing undesirable toxic effects (Hirt and Body-Malapel, 2020). Due to the EPR effect, the clearance of NPs rates from circulation streams and tissues is much lower, and it would result in the accumulation of NPs at the surface of the tissue or within cells and pose the risk of disruption of organelle integrity or gene alterations (Gitipour et al., 2016). The toxicity of NPs is highly correlated with their complexity and diversity in terms of size, shape, charge, production methods, chemical composition, surface functionalization, and aggregation (Lewinski et al., 2008; Lv et al., 2021; Nitti et al., 2022). In order to improve inorganic NPs safety, organic protective layers or coating agents are usually used to cover their surface, which would help to prolong the circulating half-life and minimize the reticuloendothelial system uptake (Nie, 2010; Rabie et al., 2019). Recent study found that when AuNPs are coated with ferritin, their biocompatibility is dramatically enhanced as the cellular uptake of transferrin-receptor-rich cell lines increased more than seven times and showed very low toxicity on different human cell lines (Moglia et al., 2021). It was reported that biocompatibility of graphene oxide could be enhanced by PEG, PAM, and PAA (Xu et al., 2014; Xu et al., 2016).

### 2.4 Targeted delivery

The lack of efficient delivery systems has impeded the efficiency of drugs, especially for small molecule drugs for chemotherapy which are always accompanied by noticeable side effects (Mu et al., 2020). In order to take the best advantages of drugs, various materials such as lipids, polymers (Go and Leal, 2021), hydrogels (Lengert et al., 2017), metal-organic framework (MOF) (Zhou et al., 2021; Kiadeh et al., 2021) and NPs (Jun et al., 2008; Xie et al., 2012; Biju, 2014; Tarakanchikova et al., 2020) have been exploited. Also recently, SiO_2_ (Gupta et al., 2018), Fe_3_O_4_ (Liu et al., 2020b), CaCO_3_ (Huang et al., 2022), and AuNPs (Sharifi et al., 2019) have been investigated extensively as emerging delivery carriers. Especially, AuNPs have many advantages, such as simple synthesis, easily tunable size, chemically inert, oxidation-free, facile surface modification, and versatile conjugation with biomolecules (Kang et al., 2019). Organic molecules can be used for functionalization of AuNP targeting specific drug delivery routes with the LbL method. For example, Lee *etc.*, designed the nanocomplex-AuNPs/siRNA/polyethyleneimine (PEI)/hyaluronic acid (Ha) modified by cysteamine, which could target specific intracellular delivery of siRNA *via* Ha receptor-mediated endocytosis (Lee et al., 2011). CaCO_3_ has proved to be useful for intracellular delivery through its crystal phase transition (Parakhonskiy et al., 2013). Recently, sub-micro vaterite CaCO_3_ particles loaded with photosensitizer drug porphyrazine (pz) was found to mainly accumulate in the tumor blood vessels, and the tumor uptake of pz was enhanced by 1.8 times; while loaded gold nanorods improved delivery by 3.4 times, showing extensive potential to be explored as tumor therapeutic agent for photodynamic therapy (Parakhonskiy et al., 2021).

### 2.5 Detection

**FIGURE 6:**
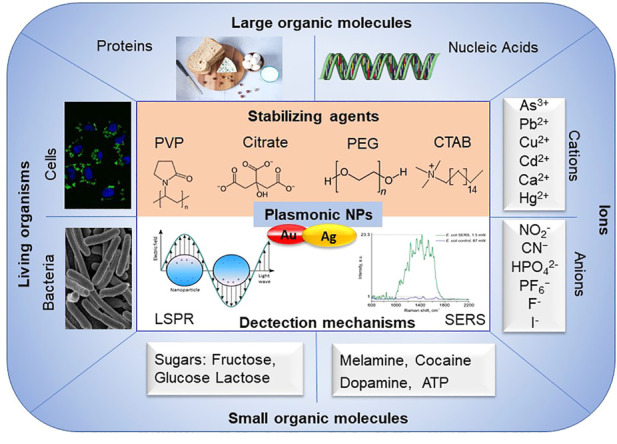
A schematic overview of mechanisms for plasmonic NPs-based biosensors for detection and their applications.

Plasmonic nanoparticles like AuNPs and AgNPs, exhibit distinct colours throughout the visible and near-infrared regions due to their localized surface plasmon resonance (LSPR) absorption and scattering (Mulvaney et al., 1992; Qin et al., 2015; Kang et al., 2019; Cui et al., 2020), and have been used in applications in sensing, energy, catalysis, and biomedicine (Lin et al., 2016; Vita et al., 2018; Dai et al., 2021; Song et al., 2021). LSPR provides a higher detection accuracy than traditionally used surface plasmon resonance (SPR). SPR emanates from coherent oscillations of conduction electrons caused by electromagnetic radiation excitation at the surface, because the size of NPs is comparable to the mean free path of electrons in these metals, which restricts the plasmon to NPs. Besides the LSPR, plasmonic nanoparticles (for example, AuNPs and AgNPs) can be also used for surface-enhanced Raman scattering (SERS) sensing, which could dramatically improve the detection sensitivity by 10^2^ to 10^14^ times (Yashchenok et al., 2012; Lengert et al., 2018). When plasmonic nanoparticles are combined with specific aptamer, antigen-antibody recognition, enzyme *etc.*, they could be modified as a specificity detection platform (Figure 6).

Magnetic NPs possess special effectsf, for example, Yang reported a combinination of polydopamine (PDA), GE11 peptide and ultra-small superparamagnetic iron oxide NPs, GE11-PDA-Pt@USPIOs which have a high specificity for EGFR-positive tumor cells; that would be used in radio-chemo combination therapy, in which such NPs enhanced magnetic resonance imaging/photoacoustic imaging (MRI/PAI) (Yang et al., 2019). Zhu et al. (2021) explored a selective and sensitive detection platform for *Staphylococcus aureus* by coating the AuNPs with polydimethylsiloxane (PDMS) film. But, it should be noted that the plasmonic NPs would lose plasmonic functionality once NPs dissolve. So one of the most critical aspects in designing and exploring the detection tools based on the plasmonic nanoparticles is to find suitable stabilizers. Commonly used stabilizing agents and size controllers include the following molecules: cetyltrimethylammonium bromide (CTAB), citrate, PVP, PEG (Kang et al., 2019). Surface plasmon resonance imaging (SPRi) was used for label-free detection based on plasmonic NPs (Guner et al., 2017). The detection platforms based on gold and silver NPs can be used to analyze many types of samples, including small organic molecules (Liu et al., 2011), nucleic acids (Thaxton et al., 2006), proteins (Anfossi et al., 2019), sugars (Brasiunas et al., 2021), cells (Zhang et al., 2010), bacteria (Li et al., 2022a), cations and anions (Liu et al., 2011; Dutta et al., 2019), *etc*.

Besides plasmonic nanoparticles, carbon quantum dots (CQDs) represent an emerging fluorescent nanomaterial which was used in various applications, especially in chemosensing and biosensing (Ding et al., 2014; Nekoueian et al., 2019). When they are functionalized by suitable organic molecules, they can be used as a versatile detection platform that includes small bioactive molecules like Vitamin B_2_, amino acids, big molecules (DNA, RNA), enzymes, or cells. When CQDs are functionalized by folic acid, a biomarker for cancer, hybrid nanoprobes could target cancer cells due to their high affinity to folate receptors (Feng and Qian, 2018).

### 2.6 Other applications outside of biomedicine

Hybrid nanocomposites based on inorganic NPs with organic molecule modifiers have various functions covering a wide range of applications, particularly in the following sectors and applications: health sector (Byrappa et al., 2008; Kopecek, 2009; Chanana et al., 2013; Ni et al., 2020; Lavrador et al., 2021), wound healing applications (Chen et al., 2022), long-term tracking stem cell transplantation therapy *in vivo* (Xie et al., 2022), electrochemical immunosensing (Campuzano et al., 2017), optics (Shavel et al., 2004; Melnikau et al., 2016; Melnikau et al., 2018), micro-electronics (Banerjee et al., 2009), transportation, packaging (Moustafa et al., 2021), energy (Li et al., 2016; Mahmood et al., 2016; Liu et al., 2019), housing (Saba et al., 2016), catalysis (Shahmoradi et al., 2011; Liu et al., 2014; Tomai et al., 2021) and the environment (Sanchez et al., 2011). Cyclodextrins (CDxs) were reported as solubilizing agents, which could improve C60 water solubility when coated by CDxs (Ikeda, 2013).

TiO_2_ based bio-catalytic nanoparticles modified by 2, 2′-Azino-bis-(3-ethyl benzothiazoline-6-sulfonicacid) (APTES), glutaraldehyde (GLU) and laccase possess a high degradation rate for such micropollutants as bisphenol-A (Hou et al., 2014) A wide-range and fast response solid-state potentiometric pH sensors based on PDA coated carbon nano-onion (CNO) electrodes were reported (Zuaznabar-Gardona and Fragoso, 2018). PDA films endow this system with a fast response toward pH changes, while CNO is responsible for transferring the signal quickly due to its large surface area and electrochemical properties. This pH sensor could work in the range from 2.2 to 8.3. Ji et al. (2020a) found that silk fibroin (SF)/Bi_2_O_2_CO_3_-Cu_2_O nanofibers produced by electrospinning can be easily recycled and have excellent photocatalytic and antibacterial activities, and low-cytotoxicity, which paves the way to promoting their practical applications in, for example, water treatment. This is because SF has a large surface to immobilize powder Bi_2_O_2_CO_3_-Cu_2_O photocatalysts, and the hybrid mat-like structure separates itself from water efficiently. The nanocomposite coating – polyurethane (PU)/SiO_2_-Al_2_O_3_ - showed significant resistance against corrosion compared to that of pure steel (Xavier, 2021).

## 3 Inorganic surfaces functionalized by organic molecules

**FIGURE 7:**
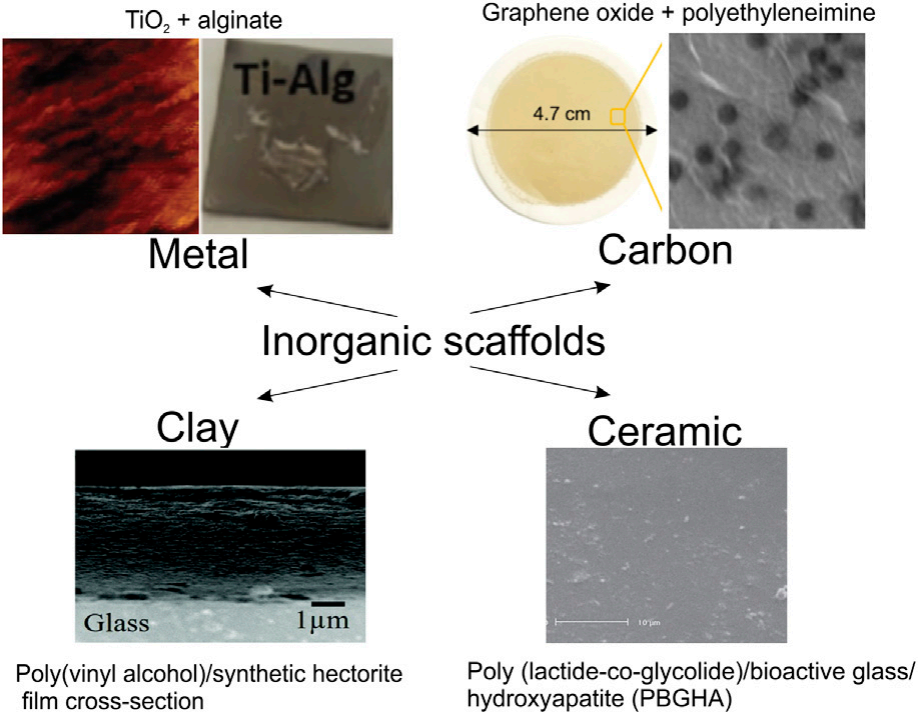
Functionalization of inorganic surfaces by organic molecules. Metal (AFM images of TiO_2_ coated by alginate hydrogel coatings reproduced from (Muderrisoglu et al., 2018) with permission Wiley-VCH), Carbon (optical and SEM images of the graphene oxide modified by polyethyleneimine film; reproduced from (Nam et al., 2016) with permission of ACS), Clay (SEM images of the Poly(vinyl alcohol)/synthetic hectorite film cross-section with a thickness of 4.3 μm (Teepakakorn and Ogawa, 2021) with permission of the Mater. Adv.), Ceramic (SEM images of the poly (lactide-co-glycolide)/bioactive glass/hydroxyapatite (PBGHA) (Mehdikhani-Nahrkhalaji et al., 2015) reproduced from Dent. Res. J. (Isfahan)).

Typically in the field of biomedicine, organic coatings provide the same functionalities as those described above for colloids, except for such applications as targeted delivery, which involves the movement of an inorganic object by itself. In Figure 7, selected inorganic surface functionalization modalities *via* organics are shown. Table 3 summarizes modifications of representative inorganic surfaces by organics highlighting their applications and gained functionalities. In the following part, the role of organic molecules is illustrated mainly for metallic and inorganic (non-metallic) materials.

**TABLE 3:** Overview of hybrid organic films/coatings on inorganic substrates/flat surfaces, their composition and functionalities.

Hybrid flat surface composition			Functionalities and applications
Inorganic flat surface		Organic films/coatings and reference	
Alloy	SS-Ti Stent	Phosphorylcholine (PC) (Huang et al. (2014b)	Load and release drugs by stimulation corresponding
	Co–Cr Stent	BioLinx (hydrophobic C10,hydrophilic C19, polyvinyl pyrrolidone) (Huang et al. (2014b)	
		PC Huang et al. (2014b)	
	Pt–Cr Stent	PLGA, CE approved	
	SS stent	Polyethylene-co-vinyl acetate (PEVA) FDA approved	Load and deliver drugs, improve the hemocompatibility, modify cells adhesion, proliferation, and migration behaviour, improve stainless steel (SS) stents extrusion resistance
		Polydopamine (PDA\) Lee et al. (2007); Yang et al. (2012)	
	Ni-Ti Stent	anti-CD34 antibody Sun et al. (2021a)	Better blood compatibility, Promote cell migration, adhesion and proliferation
		Vascular endothelial growth factor (VEGF) Sun et al. (2021a)	
		PDA Sun et al. (2021a)	
	Ti-6Al-4Vscaffolds	PDA Li et al. (2015)	
	2024 Al alloy	PEI/PSS, inhibitor (benzotriazole) Zheludkevich et al. (2007)	Active corrosion protection, self-healing property
	AZ91 Mg alloy	3-glycidoxypropyl)-trimethoxysilane (GPTMS) (Chen et al. (2020), poly (ethylene imine) (PEI)/poly (acrylic acid) (PAA), fluoropolymer (poly (vinylidene fluoride), PVDF) Zhang et al. (2021a)	
Ceramic	Bioactive glass/ hydroxyapatite (HA)	Poly (lactide-co-glycolide) Mehdikhani-Nahrkhalaji et al. (2015)	Realize tunable and sustained release of growth factors or drug), improve mechanical integrity, improve targeted cells attachment, proliferation, and bioactivity
	Bioactive glass	Chitosan Uskoković and Desai (2014)	
	Hydroxyapatite (HA)	PDAM Cai et al. (2014)	
		PDA/PLL Han et al. (2019)	
	Perovskite	PEG Fu et al. (2019)	Improve the stability of perovskite solar cells
	CaCO_3_	Alginate hydrogel Muderrisoglu et al. (2018)	Loading active enzyme
Clay	synthetic Na + -saponite (Kunimine Industries Co. Ltd.)	Sodium polyacrylate Tetsuka et al. (2007)	Enhance the thermodynamic stability, transparency of clay films
	Synthetic saponite, stevensite	Functional polymers, dye molecules, protein molecules Zhou et al. (2011)	Catalysis, modified electrodes and optoelectronic devices, anti-corrosion and packaging materials
	Iron coated montmorillonite clay (FeOx-MMT)	Gallic acid Levy et al. (2020)	Improve the surface redox reactivity
	Synthetic hectorite (SWF)	Poly (vinyl alcohol) (PVA) (Teepakakorn and Ogawa (2021)	Self-healing property, improving the adhesive ability to substrates
Carbons	Fullerene (C60)	PEG Fu et al. (2019)	Enable fullerenes to become amphiphilic
	Graphene oxide	Branched polyethene-imine Nam et al. (2016)	Enhance graphene oxide ultra thinness membranes stability
		PEI Zhang et al. (2021b)	pH-responsive corresponding, improved the hydrophilicity

^a^ SS, stainless steels; SS-Ti, stainless steels-Titanium alloys; Pt–C,r Platinum- Chromium alloys; Ni-Ti, Nickel- Titanium alloys; Co–Cr, cobalt-chromium alloys.

Functionalization of soft hydrogel-like films composed of poly-L-lysine and hyaluronic acid by inorganic nanoparticles has been shown to extend their application range (Skirtach et al., 2010; Volodkin et al., 2012), while their functionalization by capsules with nanoparticles allows to perform remote release (Volodkin et al., 2009).

### 3.1 Challenges in regard with clinical applications of biomedical materials

**FIGURE 8:**
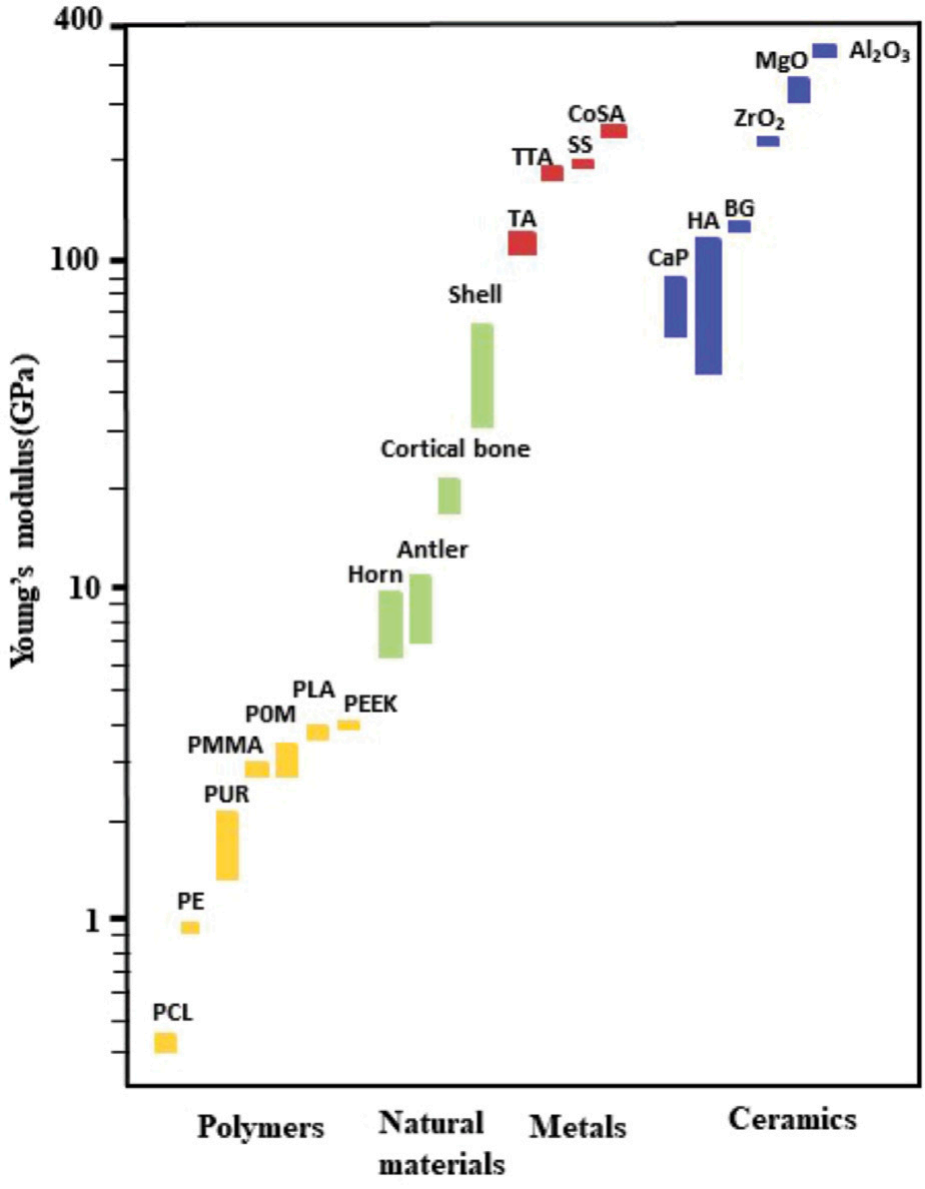
Mechanical properties (the Young’s modulus) of natural bone materials in comparison with those for other materials often used in biomedicine; data are based on (Orlovskii et al., 2002) and (Butscher et al., 2011).

Metallic biomaterials are essential for development and implementation of clinical implants to reconstruct failed tissue, especially failed hard tissue (Basova et al., 2021). Around 70%–80% of implants are made of metallic biomaterials; among which the most representative clinically used metallic biomaterials are stainless steel (SS), cobalt-chromium (Co–Cr) and titanium (Ti) alloys (Niinomi et al., 2012). Even though metallic materials have been widely used, there are still many obstacles that impede broad clincal applications of implants once and for all time, especially for long-life implantations. The common problems are mainly identified as follows (Abdel-Hady Gepreel and Niinomi, 2013):

1) mismatch of mechanical properties (Figure 8): this impedes cell attachement to the surface of the implant and leads to fibrous encapsulation at the site. Potential solution: Modification of mechanic properties and facilitation of cell attachment by organic molecules or coatings;
2) corrosion: show low fatigue strength and are vulnerable to corrosion, leading to metal ions or monomer release into the body and may damage the surrounding cell tissue. Potential solution: Application of anti-corrosion coatings;
3) lack of cell biocompatibility: allogeneic transplantation may trigger an immune response with serious side effects such as inflammation. Potential solution: Loading and covering with biocompatible polymers and growth factors.

Polymers: (PCL: poly (caprolactone), PE: polyethene, PUR: polyurethane, PMMA: poly (methyl methacrylate), POM: Polyoxymethylene, PLA: Polylactic acid, PEEK: polyetheretherketone), Metals: (TA: Titanium-based alloy, TTA: Tantalum based alloy, SS: stainless steels, CoSA: Cobalt-based super alloy), Ceramics (CaP: Calcium phosphate bio-ceramic, HA: hydroxyapatite based bio-ceramic, BG: Bioglass based bio-ceramic, ZrO_2_: Zirconia based bio-ceramic, MgO: Magnesia based bio-ceramic, Al_2_O_3_:Alumina based bio-ceramic).

### 3.2 Optimization of mechanical biocompatibility

There are two strategies to optimize the material’s mechanical biocompatibility. One strategy is towards modifying its element and structural composition from the inside. With addition of a small amount of graphene to chitosan (0.1–0.3 wt%), the hybrid graphene/chitosan films’ elastic modulus was increased by ∼ 2 times compared to that for pure chitosan (Fan et al., 2010). Taking bone repair engineering as a representative example, bone is a natural hybrid material consisting of two major parts: soft inner cancellous and hard cortical shell (Wubneh et al., 2018). Around 70% of it is composed of inorganic hydroxyapatite (HA, Ca_10_(PO_4_)_6_(OH)_2_), which is a member of the calcium phosphate (CaP) mineral family (Pal, 2014) and the rest (20%–30%) is extracellular organic matrix—a mixture of water, collagenous and non-collagenous proteins. In contrast, only around 2% is made of bone-resident cells (Belinha, 2014). Inspired by the sophisticated composite structure of bone tissue and clinical trials, the strategies of developing hybrid composites used to boost bone repair have become a hot topic.

**FIGURE 9:**
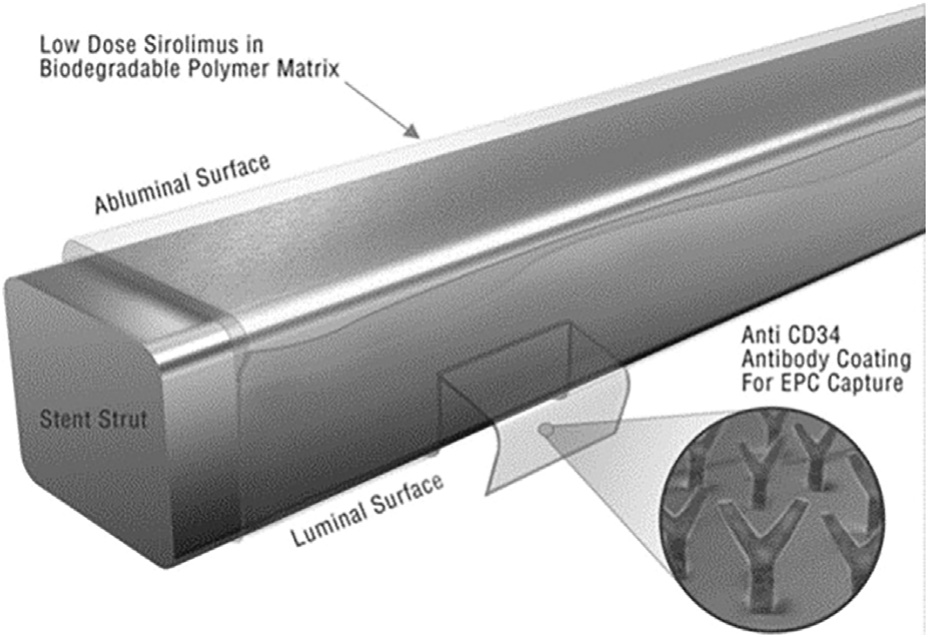
Surface functionalization of a stent by antibody coatings (Sethi and LEE, 2012) with permission of the Wiley Online Library.

The other one resorts to the surface modification and function by films or coatings to facilitate the attachment of cells to the surface of materials and to stimulate cell proliferation and differentiation (Miyazawa et al., 2008; Glazebrook et al., 2013; Ho-Shui-Ling et al., 2018). Yang et al. (2021b) found that the Ti-based implants could obtain dual function: antibacterial properties and facilitation of osteointegration *in vivo* when hyperbranched poly-L-lysine is grafted on its surface. In the area of stents, Sethi and Lee reported a successful strategy to design the combo stent where the stainless steel surface was coated by anti-human CD34 antibodies intermediated by a polysaccharide (Sethi and LEE, 2012). This combo stent showed excellent endothelial progenitor cell adhesion capability from the circulating system with the antibody coatings (Figure 9). In bone repair engineering, composites like collagen/HA, β-TCP/collagen, HA/Starch, HA/gelatin, and PCL/HA are frequently reported for surface modification (Butscher et al., 2013; Ruiz-Aguilar et al., 2017; Ho-Shui-Ling et al., 2018; Prabakaran et al., 2021). It was described that hydrogel coatings loaded with active enzymes coated on top of a titanium plate could significantly stimulate osteoblast colonization and growth (Muderrisoglu et al., 2018). It was recently shown that introduction of poly-hydrobutyrate (PHB) enables piezoresponse of CaCO_3_ mineralized 3D scaffolds, which can be used for local delivery of bioactive molecules and for enhancement of tissue repair (Chernozem et al., 2022).

Table 4 provides summary of hybrid bone scaffolds, their components, and working mechanisms.

**TABLE 4:** List of commercial products with organic bioactive molecules (growth factors, peptides or small molecules) and inorganic or hybrid material carrier and their stage of development in the bone scaffolds.

Product name (company)	Inorganic content	Organic content	Application	Stage	Mechanism of action	References
Augment® Bone Graft Wright (Medical Group)	β-TCP (powder)	rhPDGF-BB (solution)	Hindfoot and ankle fusion surgery	M	rhPDGF-BB attracts Mesenchymal Stem Cells (MSCs) to the local fusion site and stimulates its divide, proliferation as well as promotes revascularization; The β-TCP component fills the surgical defect, reliably delivers the rhPDGF-BB over time, and provides a physical scaffold for new bone formation	DiGiovanni et al. (2013); DiGiovanni et al. (2016) https://www.augmentbonegraft.com/healthcare-professionals/clinical-evidence/
Mastergraft Matrix ext, Mastergraft Strip (Medtronic)	biphasic calcium phosphate (β-TCP and HA)	bovine type 1 collagen	Bone voids or gaps filler	M	Mastergraft mimics human cancellous bone, which could be resorbed controlled and enhance osteoconductivity and vascularization without an immune response	https://global.medtronic.com/xg-en/healthcare-professionals/therapies-procedures/spinal-orthopaedic/bone-grafting/evidence/mastergraft.html
NucleostimNeovasculgen (NextGen Company Limited)	Octacalcium phosphate (OCT)	naked plasmid DNA carrying the vascular endothelial growth factor (VEGF) gene	Lumbar spine, Cervical spine, foot and ankle fusion	P- II	Plasmid DNA with VEGF gene to induce VEGF secretion by cells and promote angiogenesis; OCT as a possible precursor with extraordinary osteoconductive capacities could facilitate osteogenic cell differentiation and treated as effective scaffolds for cell delivery	NCT03076138 Bozo et al. (2021)
Amplex (Ferring Pharmaceuticals)	ß-TCP, HA	B2A (Powder)	Indicated for ankle or hindfoot arthrodesis	P- III	B2A is a synthetic multi-domain peptide augmenting osteodifferentiation *via* increasing endogenous cellular bone morphogenetic protein two by preosteoblast receptor modulation at the local arthrodesis site ß-TCP(80%), HA (20%) granules are used as a bone void filler (BVF)	NCT01224119, Glazebrook et al. (2013), https://clinicaltrials.gov/ct2/show/study/NCT03028415
i-FACTOR Bone Graft (Cerapedics)	inorganic bone mineral	small peptide P-15	Bone Graft	IDE, CE Mark	i-FACTOR Bone Grafts attract osteogenic cells, which then attach to p-15 and activate bone a natural hydroxyapatite ABM	Arnold et al. (2016), https://cerapedics.com/comparative-performance NCT01618435, NCT02895555

^a^ IDE: large human clinical study.

### 3.3 Anticorrosion protection

One of the most successful applications of coatings is anticorrosion protection. Recently, various groups developed advanced smart coatings which were made not only from the polymer layer protecting it from oxygen and aggressive ions, but also from intelligent delivery systems containing carriers with corrosion inhibitors. For example, the degradation of the organic part allows to release inhibitor and provides a self-healing effect to the damaged place (Zheludkevich et al., 2007; Deng et al., 2021).

**FIGURE 10:**
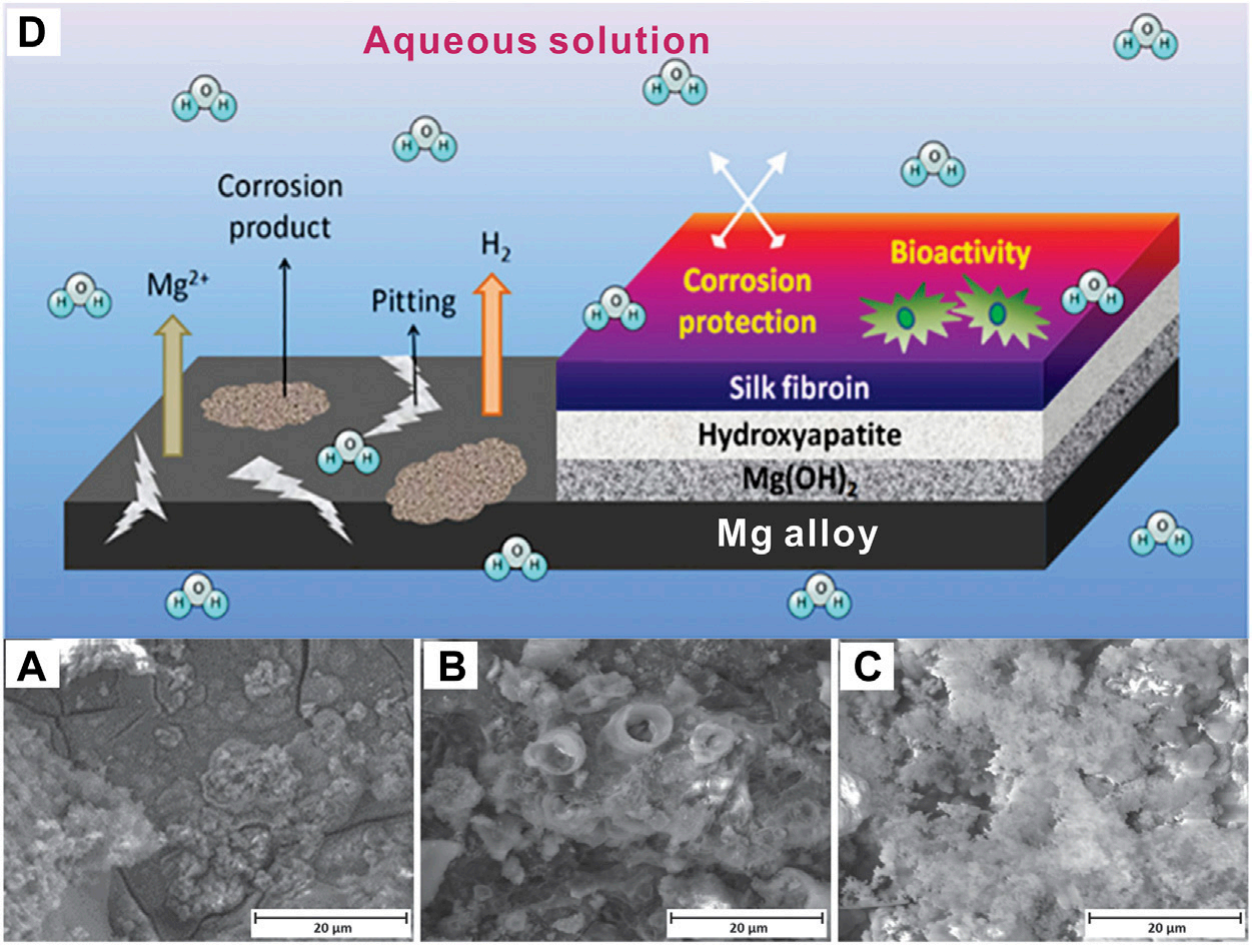
Comparison of the degradation of **(A)** pure Mg, **(B)** Hydroxyapatite on Mg, **(C)** Silk fibroin + hydroxyapatite on Mg, after immersion in Hank’s solution for 7 days, **(D)** Schematic illustration of the protection coating on Ma alloy (Rahman et al., 2021), with the permission of the ACS.

The most remarkable breakthroughs occured frequently in fully-erodible drug-eluting stents (EDES). Among the top three studied biodegradable metals (magnesium, iron, zinc), magnesium and its alloys are advancing to commercial products (Huang et al., 2014b; Zheng et al., 2014). This is because mechanical properties of magnesium are closer to the natural bone, which can dramatically minimize the stress-shielding effect–the biggest issue with non-degradable implant materials such as stainless steel and titanium alloys (Abdel-Hady Gepreel and Niinomi, 2013). However, magnesium alloys usually corrode too quickly in the human body and play a supporting role. Hybrid coatings improved their degradation resistance significantly, as Figure 10 shows (Rahman et al., 2021). Al Zoubi et al. (2020) also presented a successful strategy to enhance its corrosion resistance *via* organic coatings made up of 2-mercaptobenzimidazole (MBI). In 2016, Magmaris, a biodegradable and resorbable stent (BRS), was produced by biotronic, a German medical device company that obtained certificate approval in Europe. This remarked the first metallic BRS that became available on the market (Rapetto and Leoncini, 2017). It consists of a Mg alloy backbone and biodegradable poly-l-lactic acid (PLLA) coating, which could load sirolimus. Within the last 2 years and following-up clinical data published in May of 2021, it was reported for BIOSOLVE-IV that target lesion failure (TLF) of Magmaris is 6.6% (71/1,075 patients) and without scaffold thrombosis after 12 months of implantation, confirming its long-term safety.

### 3.4 Immune response and inflammation treatments

Organic coatings or films are explored extensively as drug and growth factor carriers to optimize implant performance and reduce or prevent the high potential risks and side effects like those mentioned above. Some representative applications are drug-eluting stents (DES), while another is bioresorable scaffolds (BRS). They comprise metal body and polymer coatings, often used in cardiovascular stents. Polymer coatings are responsible for loading and delivering drugs, such as sirolimus and paclitaxel (Garg and Serruys, 2010; Papafaklis et al., 2012; Iqbal et al., 2013). Taking three kinds of evaluated clinical Zotarolimus-eluting (ZEs) stents as a reference, a comparison between ZESs is summarized in Table 5. It can be noticed that the drug release profiles could be modified by altering the composite of metallic platforms or polymeric coatings.

**TABLE 5:** Representative ZEs comparison.

Stents brand	Platform	Polymer coating	Drug (zotarolimus) release	TLR%
Endeavour	Co–Cr	PC	95% released in half month	5.1
Resolute	Co–Cr	Hydrophobic C10, hydrophilic C19, PVP	85% elutes in 2 months	1.1
ZoMaxx	SS-Ti	PC	90% released in 1 month	9.4

^a^ TLR, target lesion revascularization at 1 year, p b 0.001 (Miyazawa et al., 2008).

**FIGURE 11:**
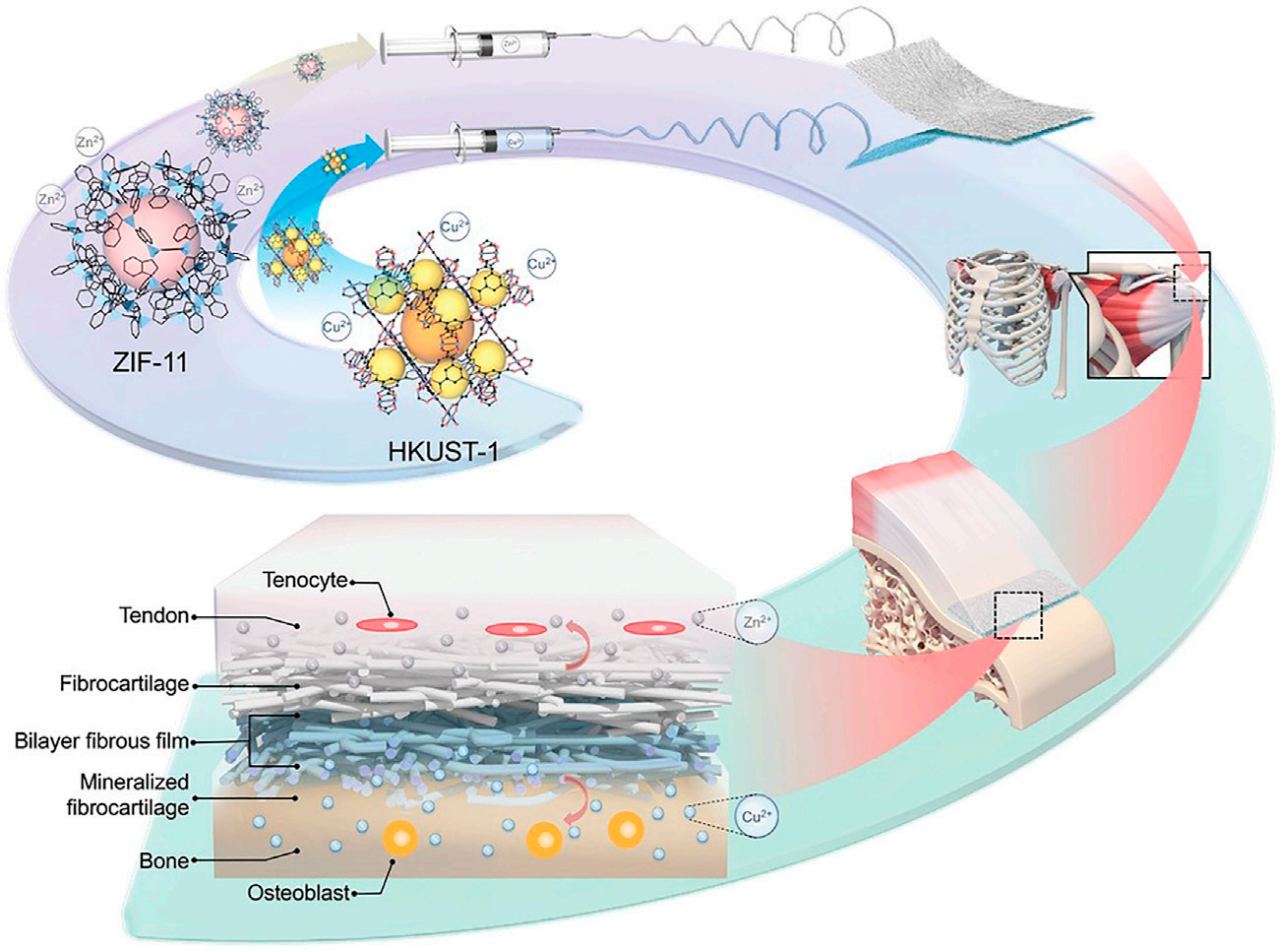
Schematic illustration of hybrid nanofibrous membrane fabrication and the effect of regulating the synchronous regeneration of the bone-tendon interface by metal ions released from the nanofiber *in situ* (Yang et al., 2022) with the permission of Wiley Online Library.

In bone repair engineering, Yang reported a successful strategy to make bipolar metal flexible fibrous membranes based on MOFs(ZIF-11 and HKUST-1), acting as carriers and achieving sustainable release of bone regeneration factors such as Cu^2+^, Zn^2+^ (Yang et al., 2022). This flexible fibrous membrane has been verified in regard with multiple tissue synchronous regeneration at the damaged tendon-to-bone interface, like tendon and bone tissue repair as well as fibrocartilage reconstruction (Figure 11).

**FIGURE 12:**
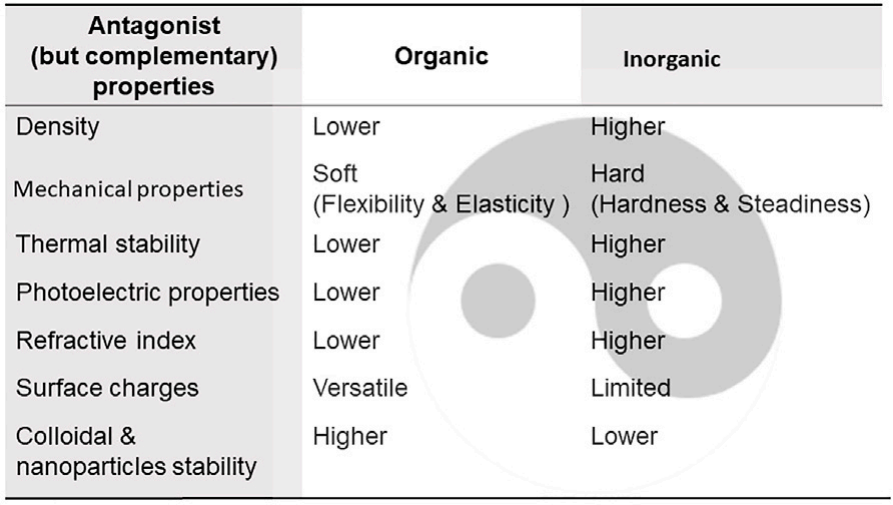
Antagonist (yin-and-yang), but complementary, properties of most common inorganic and organic compounds motivating their incorporation into hybrid materials.

Some antagonistic but complementary properties of organic and inorganic materials are summarized in Figure 12, which is useful for designing or optimizing hybrid materials with appropriate properties.

### 3.5 Other applications outside of biomedicine

Using organic films or coatings on the flat surface of the inorganics has accelerated the advancement in many areas, also outside of biomedicine. It includes protection and exploration of such materials as steel and copper, electrocatalysts (Yu et al., 2019), batteries (Li et al., 2018b; Wang et al., 2022) to advanced materials like sensors (Nugroho et al., 2018), wearable and flexible devices (Shim et al., 2008), marine ships, and aerospace vehicles (Mann, 2009; Huang et al., 2014a; Xu et al., 2021). Lee et al. (2007) reported polymer coatings synthesized from dopamine, which could coat virtually all types of material surfaces. Therefore, it could be used as a versatile platform for secondary reactions and to explore the coatings according to requirements necessitated by applications. A highly transparent flexible clay film has been obtained by organic polymer modification, which would be promising for display and other electronic devices (Tetsuka et al., 2007). A safer solid lithium battery was achieved by composite materials with polymer electrolyte (Wan et al., 2019). Teepakakorn and Ogawa reported that the poly (vinyl alcohol)-clay hybrid film has a high adhesive capability to substrates like glass and displayed self-healing soaking in water (Teepakakorn and Ogawa, 2021). Huang et al. (2019) summarized properties of organic and hybrid hydrophobic/superhydrophobic icephobic coatings for aerospace applications. PDMS-Ag@SiO2 core-shell nanocomposite antifouling coating was found to exhibit significant inhibitory effects on different bacterial strains, yeasts and fungi (Selim et al., 2018). Gu et al. (2020) reported the strategies to develop environmentally friendly marine antifouling coatings to protect ships from corrosion. The coating contains silicone polymer that prevents marine biological fouling on the ship’s surface by making it difficult for fouling organisms to adhere but facilitate removal after attachment.

Further, carbons can be functionalized with polymers. It was reported that introduction of branched polyetheneimine (bPEI) could enhance graphene oxide membranes’ stability and improve filtration efficiency (Nam et al., 2016). PEG provides fullerenes with amphiphilic properties, which enhance perovskite film quality and the perovskite solar cells’ stability (Fu et al., 2019). The utilization of PEI could make the pH-responsive graphene oxide a candidate for high-performance nanofiltration (Zhang et al., 2021b). Recently, hybrid conductive organic polymers like polyaniline, poly (dioxy-3,4-ethylenethiophene) and polypyrrole were used with traditional additive manufacturing materials like metals, paving the transition from 3D to 4D printing. Compared with traditional 3D, 4D printed material structures could response to external stimuli (voltage, force, heat), which is the core properties of soft-robotics or flexible wearable electronics (Martinelli et al., 2022).

## 4 Conclusion

The recent decades have seen a dramatic rise and extensive developments in the areas of hybrid materials and their advanced applications. Hybrid materials composed of organic and inorganic constituents can be logically divided into two parts:

1) organic materials modified with inorganic constituents (inorganics-*in*-organics),
2) inorganic materials modified or functionalized with organic molecules (organics-*on*-inorganics).

In this review, we have described and analyzed organic molecule-modified inorganic material (organics-on-inorganics) compositions and their selected applications in biomedicine and other areas. According to the size and shape of the modified inorganics, organic molecule-modified inorganic materials (organics-on-inorganics), can be sub-divided into:

a) inorganic colloids and nanoparticles functionalized by organic molecules;
b) inorganic flat surfaces/matrices functionalized by organic molecules.

Incorporation of organic constituents is selected with a specific goal—to bring or complement properties not provided by inorganics, relevant for improving their stability and biocompatibility, reducing their cytotoxicity, enhancing corrosion resistance, providing organic molecule recognition, binding specificity, detection, loading and controlled delivery, release, *etc*. And as a matter of fact, in the field of biomedicine, organic molecules provide the same functionalities for inorganic colloidal particle and inorganic flat surfaces, perhaps with exception of applications like targeted delivery. Because targeted delivery of molecules involves movement of an inorganic object by itself. More specifically, in order to clarify the synergy of organics-on-inorganics, we have illustrated the roles of organic-inorganic hybrids in various applications.

Noble metals such as gold are chemically inert, but, in addition, they possess a high photothermal sensitivity and electromagnetic conductivity. The presence of such organic constituents as polymers, antibodies or macromolecules could be used for developing and designing targeted drug delivery vehicles and achieving multi-response drug release as well as real-time *in situ* detection. For traditional non-degradable metal implants like stainless steel and titanium (Ti) alloys, the surface-coated organic antibacterial and anti-inflammation drugs could dramatically reduce the risk of secondary surgery. For degradable metals like Mg and Zn, introduction of organic polymers could be used for controlling release profile to fulfil its function and implement fully-degradable drug-eluting stents (EDES), which would safely resorb in the body. Combining MOF with organics has pushed development of highly effective multi-drug loading and multistimuli release platforms, which is achieved *via* hydrogels and amino functional groups. In the area of magnetic nanoparticles like Fe_3_O_4_, complementarity of inorganic and organic materials is achieved through dual functionality: magnetic targeting (inorganic NPs) and biocompatibility enhancement (surface functionalization by biopolymers).

Such non-metal composite-like ceramics as CaCO_3_, Ca_3_(PO_4_)_2_, HA (Ca_10_(PO_4_)_6_(OH)_2_) have been extensively used in bio-medicine. It is not only because its porous crystalline configuration provides a higher loading efficiency compared to organic carriers like liposomes, but also due to its elemental composition, which provides Ca^2+^ necessary for remodelling. Introduction of small peptides, nucleic acids, and growth factors would contribute to optimizing bone grafts and circumvent potential immune response problems. This has been tested and used in commercial bone grafts. Clays, like halloysite (Al_2_Si_2_O_5_(OH)_4_), have also attracted extensive attention due to their atomically thin layered structure and charge characteristics. Their applicability can be enhanced by adding organic molecules. For instance, in drug delivery: combining clay NPs with organic polymers, targeted proteins or polysaccharides would be useful for enhancing biocompatibility, enabling targeting, and controlling release profile. Nano carbons, like carbon quantum dots, fluorescent nanodiamonds, fullerenes, and carbon nano onion are emerging biomedical materials due to their outstanding thermal and electrical conductivity, extraordinary fluorescent brightness and photostability as well as their low density and high strength. The presence of organic molecules such as oligosaccharides, peptides, and biopolymers would pave the way for accurate imaging diagnosis.

## 5 Outlook

Although to-date developments have been very promising, innovations and breakthrough’s potential in the area of hybrid materials is yet to be fully realized. It is expected that higher levels of sophistication and miniaturization, environmental friendliness and lower production costs are expected to be achieved. Smart hybrid materials and devices will be further designed, synthesized and assembled showing excellent stimulus responses according to environmental changes based on the complementary yin-and-yang properties of organics and inorganics.

Taking as an example micro- and nano-hybrid material applications in biomedicine, the most obvious and key points are to open the “black box” of the interactions among hybrid materials with the organisms in real working situations lies in: setting-up construction strategies to synthesize the hybrids precisely with controlled stability, good biocompatibility, and excellent stimuli-responsiveness to various stimuli These smart hybrid materials would lead explorations toward such multifunctional platforms as soft-robot sensors *in vivo*, electrocatalysis, photoelectrocatalysis, wearable devices, and so on. Hybrid materials comprising organic coatings on inorganic materials are indispensable to combat modern threats such as antimicrobial resistance and nosocomial infections in general. The hybrid structure can endow the contact surfaces with specific (bio) functionalities keeping their mechanical integrity at a high level and, at the same time, providing controlled presentation of active compounds hosted/protected in organic part on demand.

A critical mass of interdisciplinary knowledge is needed for realization of this vision. To be more specific, the advanced characterization methods, emerging materials, and a collaboration of different research communities would dig out deep into the properties of hybrid materials and facilitate advanced materials applications.

## Glossary

α-CHC: α-cyano-4-hydroxycinnamic acid
AHAPS: N-(6-aminohexyl)-3-aminopropyltrimethoxy silane
ALP: alkaline phosphatase
APTES: 3-aminopropyltriethoxysilane
BPEI: branched polyethylene-imine
CDxs: Cyclodextrins
CHA: catalytic hairpin assembly
CNO: carbon nano-onion
CQDs: carbon quantum dots
CTAB: cetyltrimethylammonium bromide
CTS: Chitosan
DDA: dodecyl amine
DMAP: 4-(dimethylamino)pyridine
GO: graphene oxide
GLU: glutaraldehyde
Ha: hyaluronic acid
HA: hydroxyapatite
HAD: hexadecyl amine
HDL: high-density lipoprotein
HDTMA: Hexadecyl trimethy-lammonium bromide
MCT-β-CD: Monochlorotriazinyl-β-cyclodextrin
MOF: Organic Framework
MWCNTs: multi-walled carbon nanotubes
PAA: polyacrylic acid
PAH: poly(allylamine hydrochloride)
PC: Phosphorylcholine
PCL: poly(caprolactone)
PDA: polydopamine
PDDA: poly (diallyl dimethyl ammonium chloride)
PDMS: polydimethylsiloxane
PE: polyethene
PEA: poly(ester-amide)
PDMS: polydimethylsiloxane
PEEK: polyetheretherketone
PEG: poly(ethylene glycol)
PEI: polyethyleneimine
PEO: poly ethylene oxide
PEVA: Polyethylene-co-vinyl acetate
PLA: Polylactic acid
PLL: poly(L-lysine)
PMDA: Pyromellitic dianhydride
PMMA: poly(methyl methacrylate)
PMPC: polymethacryloyloxyethyl phosphorylcholine
POM: Polyoxymethylene
PSS: poly(sodium styrenesulfonate)
PU: Polyurethane
PUR: Polyurethane
PVP: polyvinyl pyrrolidone
PVDF: poly (vinylidene fluoride)
SPA: polyacrylate
SS: stainless steels
TOPO: trioctylphosphine oxide
